# An Investigation of the Energy Harvesting Capabilities of a Novel Three-Dimensional Super-Cell Phononic Crystal with a Local Resonance Structure

**DOI:** 10.3390/s24020361

**Published:** 2024-01-07

**Authors:** Hang Xiang, Zhemin Chai, Wenjun Kou, Huanchao Zhong, Jiawei Xiang

**Affiliations:** 1 School of Mathematics and Computer Science, Northwest Minzu University, Lanzhou 730106, China; p211513443@stu.xbmu.edu.cn; 2 College of Mechanical and Electrical Engineering, Wenzhou University, Wenzhou 325035, China; 21451439002@stu.wzu.edu.cn; 3 Experimental Teaching Department, Northwest Minzu University, Lanzhou 730000, China; 185072075@xbmu.edu.cn

**Keywords:** energy harvesting, locally resonant phononic crystals, super-cell, finite element method, piezoelectric effect, band gaps

## Abstract

Using the piezoelectric (PZT) effect, energy-harvesting has become possible for phononic crystal (PnC). Low-frequency vibration energy harvesting is more of a challenge, which can be solved by local resonance phononic crystals (LRPnCs). A novel three-dimensional (3D) energy harvesting LRPnC is proposed and further analyzed using the finite element method (FEM) software COMSOL. The 3D LRPnC with spiral unit-cell structures is constructed with a low initial frequency and wide band gaps (BGs). According to the large vibration deformation of the elastic beam near the scatterer, a PZT sheet is mounted in the surface of that beam, to harvest the energy of elastic waves using the PZT effect. To further improve the energy-harvesting performance, a 5 × 5 super-cell is numerically constructed. Numerical simulations show that the present 3D super-cell PnC structure can make full use of the advantages of the large vibration deformation and the PZT effect, i.e., the BGs with a frequency range from 28.47 Hz to 194.21 Hz with a bandwidth of 142.7 Hz, and the maximum voltage output is about 29.3 V under effective sound pressure with a peak power of 11.5 µW. The present super-cell phononic crystal structure provides better support for low-frequency vibration energy harvesting, when designing PnCs, than that of the traditional Prague type.

## 1. Introduction

Self-powering wireless microsensors are a key device in the era of the Internet of Things (IoT). Effectively converting mechanical energy into utilizable electrical energy has attracted the attention of a wide range of scholars. Due to their studies, environmental mechanical energy, e.g., the energy manufactured by vibration waves and acoustic waves (both belong to elastic waves), during the transmission process, can be transformed into electric power using the piezoelectric effect. Therefore, coupling piezoelectric materials with acoustic metamaterials to obtain complete energy-harvesting systems can be utilized to continuously supply electricity for wireless microsensors, etc. [1,2,3,4,5]. These systems not only resist vibration and reduce noise, but harvest wave energy to achieve the objective of energy self-sufficiency. Since diverse PnC structures demonstrate distinctive material phenomena and features, these structures can be actualized to manipulate the efficiency of elastic waves using the piezoelectric effect [6,7,8]. The coupling system provides a useful exploration method for vibration and sound suppression, acoustic hypersensitivity, energy location identification and the harvest of the elastic wave. On account of the research interests previously mentioned, numerous PnC structures are proposed in this article to improve energy-harvesting efficiency [9,10,11].

The structures investigated here stem from the physical mechanism of local resonance, which have an edge on traditional Bragg scatter types [12,13,14,15]. Typically, the BGs of Bragg scatter types generally have high frequencies. Xu et al. obtained a structure with a Bragg scatter PnC that had a frequency of 120 kHz [16]. Li et al. explored structures with high-frequency BGs but a wide bandwidth, which were Bragg scatter types [17]. Meanwhile, the research direction on PZT energy harvesting of Bragg scatter type is in the defect mode. Yang et al. expounded the broadband characteristics of high-performance energy-harvesting PnCs with point defects [18]. Defect mode analyses of PnC structures were carried out by Nagaty et al., enabling the development of super-cell generated sensors, for applications in the area of magnetic measurement [19].

This early research on PnC structures verified that the Bragg scatter type PnC can be potentially used to drive microsensors. However, achieving the target of self-sufficiently-powered wireless sensors by harvesting low-frequency vibration energy is still a recognized difficulty. Throughout the development process of acoustic metamaterials, the local resonance mechanism set up a new route of extending research into low-frequency vibration energy harvesting structures. Zhai et al. optimized the geometric parameters for a 2D locally resonant phononic crystal with a toroidal structure that obtained the lowest starting frequency, whose corresponding BG was 20 Hz [20]. Moving from a 2D model to a 3D model, Ma et al. developed a 3D PnC with a local resonance windmill-like structure, with two corresponding BGs of 87–125 Hz and 166–247.8 Hz [21]. When LRPnC unfold band gaps, the characteristic of local resonance causes the maximum deformation of structure near the BGs. In view of the mechanism mentioned above, Shen et al. put forward a locally resonant phononic crystal plate with low-frequency vibration energy harvesting, with a frequency of BG of 29.2 Hz to 65.8 Hz. While having such a low BG range, Shen et al. formed a series circuit through several super-cell crystals, resulting in a voltage of 18 V [22]. However, though a comprehensive survey of those structures, we can conclude that, whether we use the Bragg scatter type PnC with point defects or the circuit series connection with local resonance, the voltage of the energy harvesting is still insufficient [23,24].

The structure proposed in this work aims to have an optimal interval of BGs and augment the voltage generated by LRPnCs. LRPnCs possess excellent low-frequency bandgap properties. Moreover, some flat bands appear in the wave field when the bandgap is open. Under the excitation of the corresponding frequency of the flat band, the LRPnC vibrates locally, and the energy is gathered inside the unit-cell of the LRPnC. Also, the existence of flat bands implies that the wave propagation pattern is identical in most wave vector directions, under the excitation of the corresponding frequency. These two conditions provide a new approach to low-frequency energy harvesting using LRPnCs [25,26]. Through these previous studies of locally resonant energy self-sufficient PnCs, a 3D super-cell PnC with local resonance spiral structure is investigated by COMSOL, using FEM to build a physical field control grid; thus, parameterized scanning in specified combinations is performed subsequently. In general, BGs and vibration shapes should be regarded as prominent impact factors for energy harvesting, so the maximum structure deformation and BGs are obtained from the vibration mode. Through the characteristics of this structure, the BGs near plasmon bands and the maximum deformation point are analyzed to acquire optimal coupling position for PZT sheet. However, the following problem is still to be solved: the vibration modes of each unit-cell are distinctive and not suitable for the constructed super-cell form [27]. Consequently, we make a comparison with the vibration modes in dissimilar structure frequencies, then the maximum deformation point attached to the beam in one of the unit-cells is revealed, which is then interpreted in detail below. After resolving this matter, the coupling relationship between the PZT sheet and the maximum deformation point are established, whereupon the energy recovery circuit of each single unit-cell is connected to the PZT sheet. Finally, after the analysis of a multi-field physics simulation based on the piezoelectric effect, a high voltage recycling structure with low BGs will be obtained. Therefore, the main contributions of this work are summarized as follows:

- Constructing a structure for subsequent coupling with piezoelectric effects by changing the geometric parameters and the location of beams.
- Implementing the broadband characteristics of high output power by depositing the PZT sheet on the maximum deformation point.

In the remaining part of this article, the assay of unit cell 3D PnC structure is conducted in Section 2. In Section 3, the initial frequency, bandwidth and vibration modes of BGs are obtained. In Section 4, the energy-harvesting simulation is calculated to obtain the voltage and power numerical number. Eventually, conclusion remarks are given in Section 5.

## 2. Construction of a Unit-Cell for Three-Dimensional Phononic Crystals with a Local Resonance Spiral Structure

The finite element simulation in this paper is implemented by the software COMSOL Multiphysics 5.6. The advantages of the FEM lie in its ability to solve partial differential equations and integral equations in complex structures in both frequency and time domains, as well as in its clear and simple conceptualization of the computational methods, good convergence, fast adaptability and high computational speed. In particular, the commercial finite element software COMSOL Multiphysics is widely used in the computation of applied models of 2D and 3D PnCs because of its ability to deal with complex multi-physics field coupling problems, to provide advanced mesh generation and post-processing tools, and to generate high-quality simulation results.

Before analyzing the overall structure, the detailed description of the assembly process is provided in this chapter. The principle of the locally resonant type is different from the Bragg scatter type, because when the frequency of the elastic waves propagating in the matrix approaches the resonant frequency of the unit-cell, the resonant structural unit will have a strong coupling effect with the elastic wave, preventing it from continuing to propagate forward, resulting in the generation of a band gap. A unit-cell of a 3D PnC with a local resonance spiral structure is created, in which the elastic beams constitute a helical conformation.

While calculating the band structure of the unit cell, due to the periodicity of the PnC, the Bloch–Floquet theorem is performed at the ideal crystal, and one unit-cell boundaries are set with periodic conditions, as represented by Equation (1) [28]:

(1) $$\psi (r+a)=e^{i(k\cdot a)}\Psi (r)$$

The physical quantities in Equation (1) are interpreted below. Ψ is the phase shift, *r* is the variable located at the boundary, *a* is the lattice period vector and *k* is the wave vector. The Bloch wave vector *k* = (*kx*, *ky*) on the boundary is defined by the Bloch periodic boundary condition. The wave vector *k* on the first Brillouin curve establishes the dispersion curve in the propagation direction. For the X direction, following the scanning of the irreducible Brillouin zone Г-X-M-Г, the BG properties and intrinsic modes of the PnC structure are derived, and the wave vector is signified by *k*. Even if the formula is built on the basis of one unit cell, the periodic conditions do not excessively constrain the resonance of it, which can also be applied to a super-cell. In Section 3, a detailed analysis on the BGs of the super-cell will be provided accordingly.

**Figure 1:**
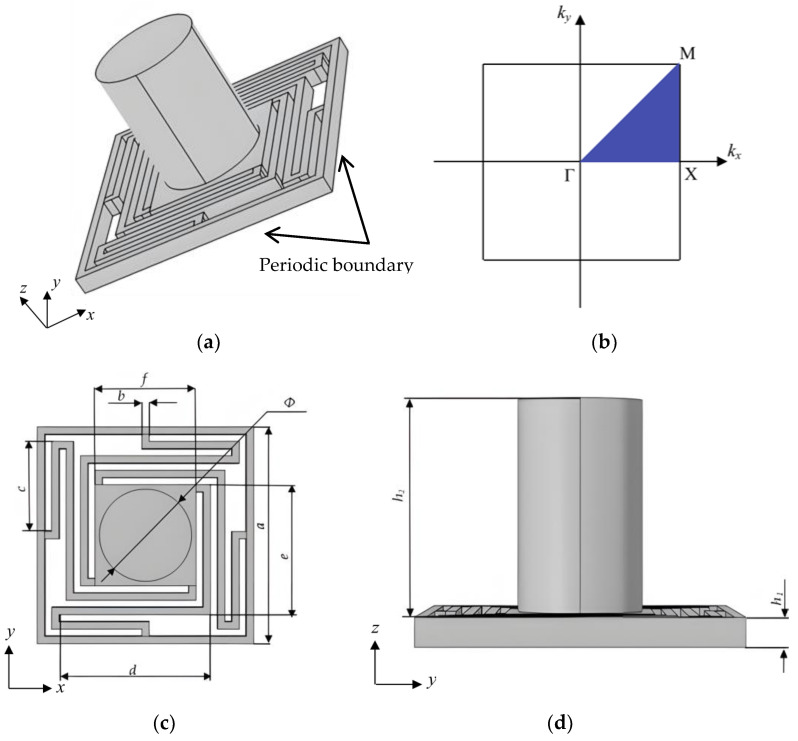
(**a**)The unit-cell, (**b**) the irreducible first Brillouin zone, (**c**) the front view of the unit-cell and (**d**) the plane view of the unit-cell.

As shown in Figure 1, the structure model is a square lattice unit-cell, consisting of a scatterer surround by four helical elastic beams. The dimensions of structure are as follows: within the X–Y axis, *a* is the lattice constant of the unit-cell, *b* is the unique thickness of the elastic beams, *f* is the side length of the square scatterer, *Φ* is the diameter of the additional weight blocks for the scatterer and *c*, *d* and *e* are the lengths of each elastic beam, respectively. As shown in Figure 1d, *h*_1_ and *h*_2_ are the thickness of the frame and the height of the weighted cylinder, respectively.

In the FEM simulations, the geometric parameters of the 3D structure are as follows: *a* = 30 mm, *b* = 1 mm, *c* = 12.5 mm, *d* = 21 mm, *e* = 18 mm, *f* = 14 mm, *Φ* = 12 mm, *h*_1_ = 2 mm and *h*_2_ = 15 mm. The material of the elastic beam and frame is PA6, the scatterer and the weighted cylinder are aluminum metal. The mechanical parameters of these two materials are presented in Table 1.

**Figure 2:**
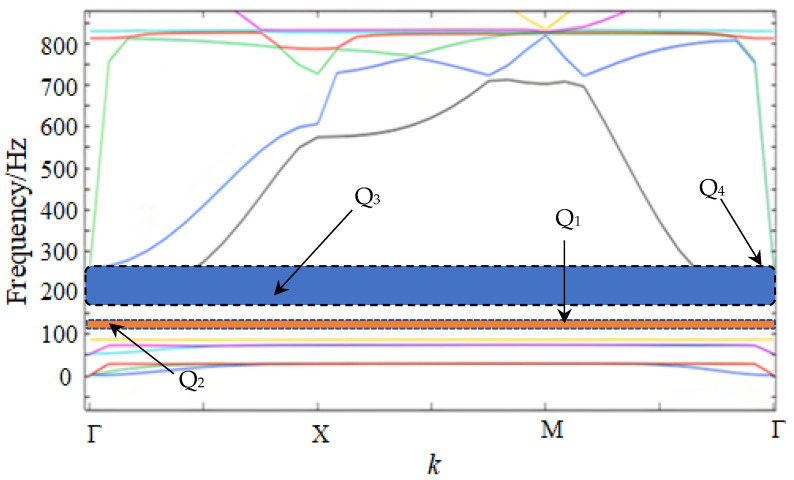
The diagram of low-frequency band gaps.

Generally, the ratio of density and Young’s modulus determines the low-frequency BGs. Due to the finite period limitations from the super-cell structure, the unit-cell is analyzed to verify the periodicity pattern. Fabricating the mesh based on FEM using COMSOL, the characteristic of the unit-cell is calculated, and the band spectrum is drawn. In Figure 2, the spectrum points out that the starting frequency is Q_1_ and the cut-off frequency is Q_2_, and the details of the boundaries Q_1_, Q_2_, Q_3_ and Q_4_ are 28.37 Hz, 51.34 Hz, 73.02 Hz and 193.55 Hz, respectively. The relationships between the displacement mode shapes and the boundary points Q_1_, Q_2_, Q_3_ and Q_4_ are delivered. Through the below diagram, the wave vector *k* displayed at each boundary point from Q_1_, Q_2_, Q_3_ and Q_4_ are 2, 0, 1 and 3, respectively. Based on the fundamental features of locally resonant PnCs, the boundary point is the limit of the coupling between the elastic waves and the structural elements.

**Figure 3:**
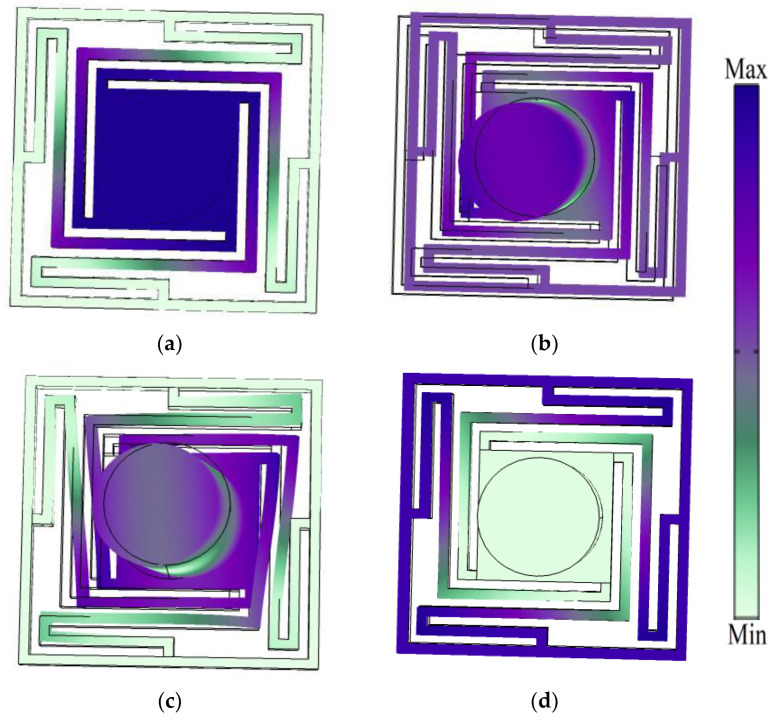
The displacement mode shapes of the unit-cell: (**a**) Q_1_, (**b**) Q_2_, (**c**) Q_3_ and (**d**) Q_4_.

As seen in Figure 3, the displacement mode shapes (a), (b), (c) and (d) of the unit-cell represent the boundary points Q_1_, Q_2_, Q_3_ and Q_4_, respectively. According to the displacement mode shapes, the deformation degree in elastic beams is weak and the mass of the scatterer contributes the bulk of the mass on the coupling between the elastic wave and the structure.

As seen in Figure 3, mode Q_1_, with 28.37 Hz, shows that the displacement response is concentrated in the mass block and contiguous elastic beams; despite this, the frame still maintains a stable state. In contrast to mode Q_1_, mode Q_2_, with 51.34 Hz, displays parallel displacement in the overall unit-cell structure, and vibration reactions are prominent throughout the whole construction. Then, in mode Q_3_, with 73.02 Hz, the coupling relations obviously occur in the elastic beams near the scatterer. Finally, the displacement mode shape in the Q_4_cut-off frequency boundary point confirm that the vibration response occurred in the elastic beams away from the scatterer.

After examining the above unit-cell data, it is clear that the band spectrum and displacement mode shapes of the unit-cell are not an effective basis to infer the characteristics of a super-cell structure, but the basic appearance of the BGs of that super-cell structure has emerged. In the light of these data, a 5 × 5 super-cell structure with homologous 3D PnCs is constructed, to conduct energy harvesting.

## 3. Analysis of 5 × 5 Super-Cell Three-Dimensional Phononic Crystals with a Local Resonance Spiral Structure

**Figure 4:**
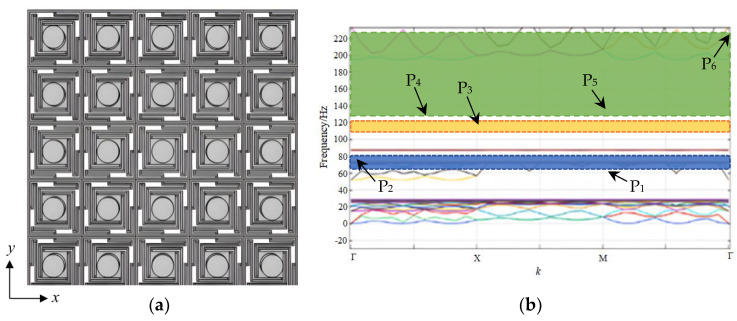
(**a**) the 5 × 5 super-cell 3D PnC, (**b**) the BGs of super-cell.

Following the fundamental investigation in Section 2, a super-cell 3D PnC with a local resonance spiral structure is proposed in this section. With its construction based on the unit-cell proposed in the above chapter, the super-cell 3D PnC is designed as a 5 × 5 homologous 3D PnC, which takes into account the actual application scope. Though the finite periodic LRPnCs made into super-cells are considered to not be not particularly dependent on periodicity [29], the point defect in super-cell LRPnCs have little impact on the piezoelectric recovery experiment. Therefore, the 5 × 5 super-cell PnC with a local resonance spiral structure still compares with the unit-cell PnC, which may obtain different BGs. The overall scale of the super-cell 3D PnC is 150 mm × 150 mm, and the boundaries of each cell are closely connected to compose a finite periodic structure. Figure 4. shows the entire structure of 5 × 5 homologous 3D PnC.

According to the diagram of BGs shown in Figure 4, the structure of the 5 × 5 super-cell 3D PnC obtains more complex plasmon bands than those of the homologous unit-cell. The distinction between the super-cell and the unit-cell is obviously the number of boundary points, which has changed from four to six. Intricate designs show that P_1_, P_2_, P_3_, P_4_, P_5_ and P_6_ are 28.47 Hz, 51.44 Hz, 73.71 Hz, 86.59 Hz, 87.41 Hz and 194.21 Hz. In the unit-cell, no other plasmon bands are distributed between the boundary bands, which are located in boundary points P_3_ and P_4_. Then, in the 5 × 5 super-cell 3D PnC, multiple plasmon bands split the boundary bands between 73.71 Hz and 194.21 Hz, manufacturing other two boundary bands; therefore, a new BG is formed. In other words, the plasmon bands with frequencies from 86.59 Hz to 87.41 Hz are regarded as defect bands. In theory, the defect bands actually have little impact on piezoelectric recovery systems for locally resonant PnCs.

**Figure 5:**
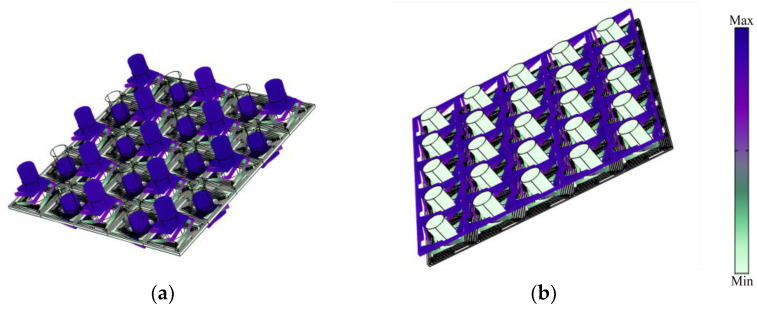
The displacement mode shapes of 5 × 5 super-cell 3D PnCs: (**a**) P_1_ and (**b**) P_6_.

After the analysis of the BG characteristics, the displacement modes P_1_ and P_6_ are plotted in Figure 5. P_1_ and P_2_ are the initial frequency band and terminal frequency band, respectively. As seen in Figure 5a, the displacement of the first starting frequency results in symmetrical distribution. In Figure 5b, the displacement of the third cut-off frequency results in the beams of the structure exhibiting an integral upward trend. From mode P_1_ to mode P_6_, the displacement mode shapes reflect the characteristics of orderliness and symmetry, which are conspicuous. As seen in Figure 4 and Figure 5, the wave vector *k* from boundary points P_1_ and P_6_ is 2 and 3, respectively; it follows that the BGs opened by the 5 × 5 super-cell structure, due to resonance, are similar to the homologous unit-cell. Therefore, the localized resonant PnCs with finite periodic structures are still suitable for periodic conditions.

**Figure 6:**
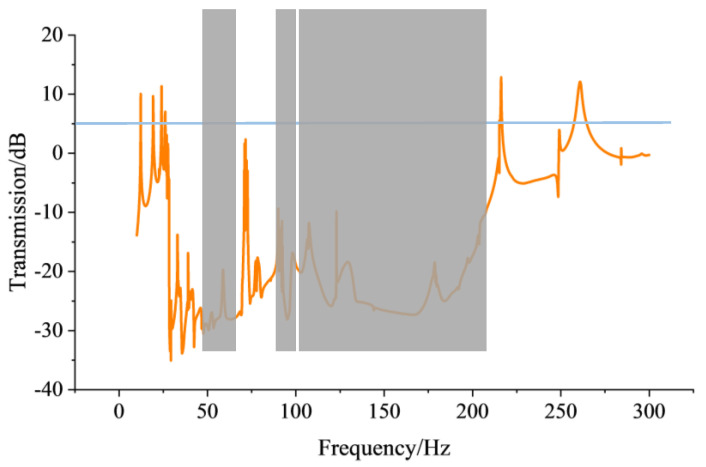
The transmission spectrum of the 5 × 5 super-cell 3D PnC.

Before implementing energy harvesting architecture into the model, the transmission relation of the elastic waves is calculated with line integration on the 5 × 5 super-cell 3D PnCs and the corresponding transmission spectrum is plotted, as shown in Figure 6. From the transmission spectrum, it is simple to make a distinction between attenuation and enhancement, and the gray regions are defined as the parallel frequency range, as seen in Figure 4b. As seen when observing the gray regions of the transmission spectrum, the vast majority of the elastic waves at corresponding BG frequencies indicate attenuation characteristics when they are propagated through the 5 × 5 super-cell construction. Moreover, the attenuation areas stem from complex pass-bands generated by the finite periodic structure. In summary, a positive consensus is reached between the frequency range corresponding to gray regions and the band structure, as shown in Figure 4.

When examining the data from the unit-cell and the 5 × 5 super-cell 3D PnCs, we can see that the periodicity of this LRPnC is not completely disrupted, and that the defect modes have a low effect on transmission. Therefore, the global data illustrate that the BG disparity between the 5 × 5 super-cell and one unit-cell is minute, except for defect bands. Consequently, the 5 × 5 super-cell 3D PnC with a local resonance spiral structure is employed to construct the energy harvesting system. Beyond that, the location of the maximum vibration deformation is confirmed by the displacement shape modes corresponding to each boundary frequency, so the PZT sheet is ensconced on the prescriptive beam. After confirming the accurate placement position for the PZT sheet, the coupling algorithm for the physical field is employed, to obtain the voltage value generated by the 5 × 5 super-cell 3D PnC.

## 4. Numerical Simulations

**Figure 7:**
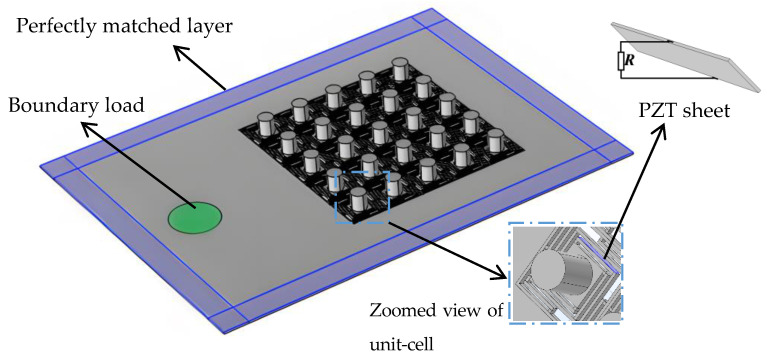
The structure of the energy harvesting system.

In this section, a novel energy harvesting system, consisting of the 5 × 5 super-cell 3D PnC with a local resonance spiral structure, is proposed. Based on the piezoelectric effect, the system achieves energy harvesting through the propagation of elastic waves. As shown in Figure 7, the 5 × 5 super-cell 3D PnC is embedded in the pedestal made of aluminum. The pedestal divides into two modules: the perfectly matched layer (PML) and the boundary load. The PML, surrounded by the 5 × 5 super-cell 3D PnC, is instrumental in observing the wave localization phenomenon under incident unidirectional plane waves, and the boundary load reflects the external force acting on the component. Furthermore, the dimensions of the whole system structure are 350 mm × 250 mm × 2 mm.

Based on the maximum vibration deformation of the 5 × 5 super-cell 3D PnC analyzed in the previous section, the PZT sheet is ensconced on the right side of the first elastic beams, near the scatterer in the homologous unit-cell, or at the bottom left corner of the 5 × 5 super-cell, as indicated in Figure 7. The scale of the PZT sheet is 17 mm × 0.15 mm × 2 mm; the thin width is to prevent the damage from mutual compressing with the elastic beams. Moreover, the external circuit of the PZT sheet is connected in series.

Considering the performance of the PZT sheet, the PZT-5H is used to constitute the core device of the energy harvesting system, because of its high sensitivity and high dielectric constant values. The following PZT-5 parameters and the parameters from Table 1 are used for the calculation of the coupled physical fields. The partial parameters of PZT-5H are introduced in Table 2.

By analyzing the characteristics demonstrated in Figure 4b, the following conclusions are drawn. The elastic waves propagate at corresponding BG frequencies, causing resonance within the structure, whereupon electric energy is generated by the PZT sheet. The scanning range of the frequency is determined to be 0–200 Hz, hence the bandwidth of the BGs, as shown in above section. The purpose of determining the scope is to give prominence to the highest point of voltage; moreover, the principle of energy recovery is based on the local resonance generated within the range of the BGs, and the piezoelectric phenomenon is extremely inconspicuous outside the BG range.

**Figure 8:**
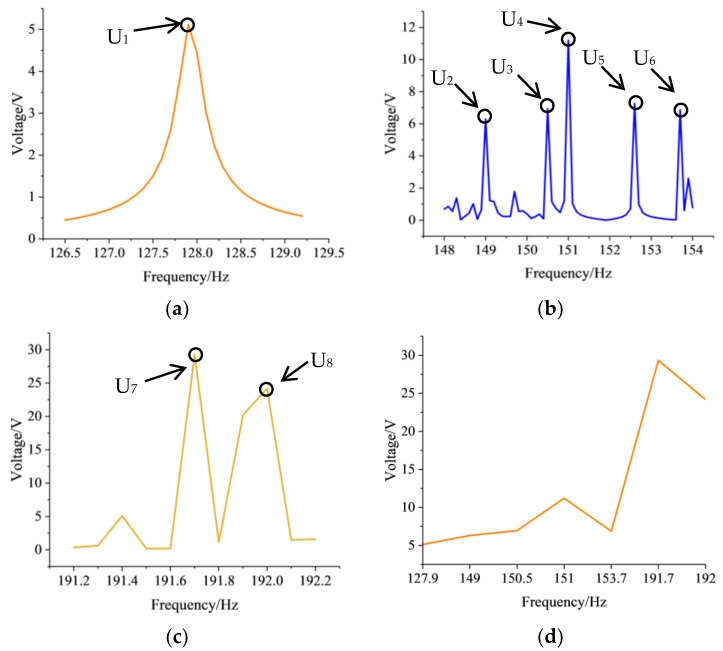
Voltage output in the frequency range of (**a**) 126 Hz–130 Hz, (**b**) 148 Hz–154 Hz, (**c**) 191.2 Hz–192.2 Hz, (**d**) 127.9 Hz–192 Hz.

When we comply with the scanning range of the frequency, the physical field is coupled with the energy recovery system, to obtain the voltage generated by piezoelectric effect. After integrating the data calculated by FEM, the relationship between the voltage and the frequency of the BGs is shown in Figure 8. Inspecting the figures below, we can see that the voltage generated during the deformation of vibration mode has been discovered, and eight outstanding high voltage points are marked. In contrast to the characteristics of the Bragg scatter type, the highest voltage points of the local resonance type occur far away from the defect bands, which proves the correctness of the analysis in previous section.

As shown in Figure 8, the marked points are distinguished as U_1_, U_2_, U_3_, U_4_, U_5_, U_6_, U_7_ and U_8_, and their corresponding frequencies are 127.9 Hz, 149 Hz, 150.5 Hz, 151 Hz, 153.7 Hz, 191.7 Hz and 192 Hz, respectively. The voltage generated by local resonance at 191.7 Hz reaches 29.34 V, which is the highest, and even the lowest voltage points, located at 127.9 Hz, amounts to 5.11 V. From a low frequency to a high frequency, the peak output voltage presents an upward trend in Figure 8d. Due to the acceleration of the vibration frequency, the resonance phenomenon leads to a more prominent piezoelectric effect. Based on the above analysis, we concluded that the local resonance type provides a significant effect on energy localization and an excellent energy output performance.

**Figure 9:**
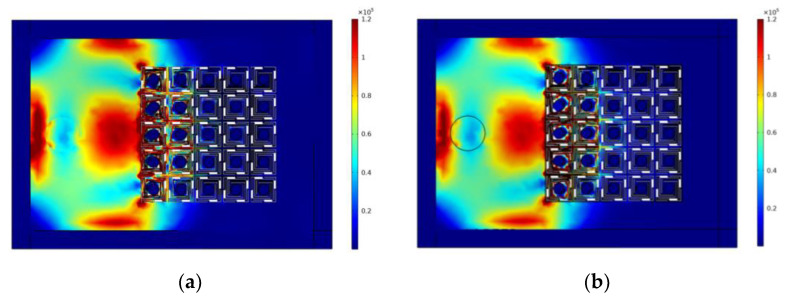
Stress distribution for frequencies of (**a**) 191.7 Hz and (**b**) 192 Hz.

The stress distribution of the device at the frequencies of 191.7 Hz and 192 Hz is also analyzed, as shown in Figure 9. From the stress distribution visualization it can be seen that, when the elastic wave reaches the super-cell, the vibrational energy is localized in the elastic beam, and the elastic wave undergoes different degrees of weakening in the subsequent propagation. These results corroborate the previous analysis.

**Figure 10:**
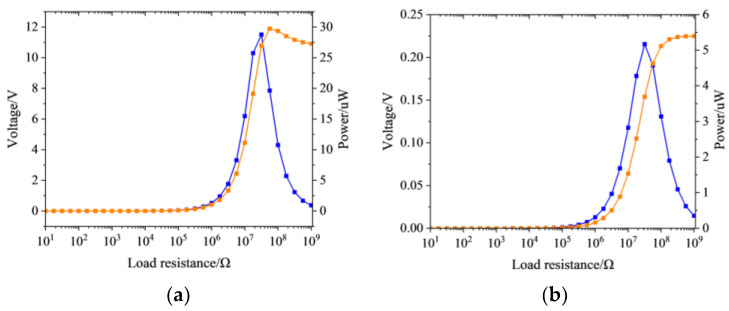
The output performance of the 3D PnC at (**a**) 191.7 Hz and (**b**) 127.9 Hz.

Based on the relationship between the peak output voltage and the BG frequency, the external resistance is loaded on the PZT sheet, to calculate the power variation curve. Two sets of peak output voltage are analyzed, including the maximum voltage point and the minimum voltage point. The load resistance is scanned from 0 Ω to 10^9^ Ω, through simulation, and the output performances are given in Figure 10.

As displayed in the illustration above, it can be clearly observed that, as the load resistance increases, the output voltage monotonically increases and converges to the peak. Under the optimal load resistance, the peak output power reaches up to 11.5 µW and 0.22 µW, separately. The optimal load resistance for the electrical power at 191.7 Hz and 127.9 Hz is 3.2 × 10^7^ Ω. Furthermore, the impedance mismatch of a piezoelectric energy recovery device, based on a local resonance PnC at 191.7 Hz, is less than one at 127.9 Hz. Furthermore, the same goes for the other peak output voltage points.

**Figure 11:**
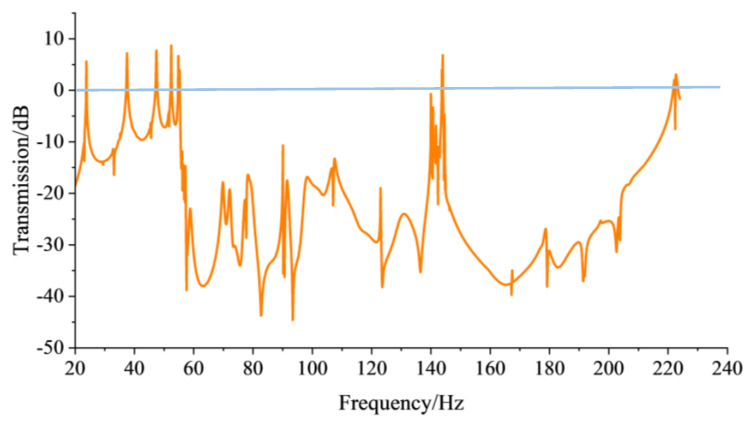
The transmission spectrum of the energy harvesting system.

The transmission relationship of the overall system is analyzed using the FEM result from the base, which contains the perfectly matched layer and the external loading domain and has an impact on vibration transmission [30]. Varying from the line integral method mentioned in Section 3, the edge surface integral algorithm is adopted for faster solving. Integrating from the edge to the interior emphasizes the impact caused by the pedestal. The calculated outcome of the transmission spectrum is shown in Figure 11.

In Figure 11, the attenuation and enhanced area can be clearly observed to be significantly different from the transmission relationship of the 5 × 5 super-cell 3D PnC. However, the peak output voltage points in corresponding frequencies are still presenting the attenuation, and a few proportions of the enhanced region locates in the BG frequencies are different from the super-cell. The reason for the abnormal phenomenon is the pedestal, which affects the regular propagation of the elastic waves in the periodic structure. Although the complex pedestal makes a difference, the transmission relationship of the whole system obtains a better reflection. 

For the energy harvesting system, the entire simulation’s experimental phenomenon is notable. The created peak output voltage, up to 29.34 V, is sufficient to verify the superiority of the system with a local resonance structure.

## 5. Conclusions

In this article, a novel local resonance phononic crystal is proposed for the application of low-frequency vibrational energy harvesting. To start with, the structure of the PnC is calculated, to obtain the low starting frequency and wide bandwidth, the corresponding first starting BG frequency of which is 28.47 Hz, and the whole bandwidth obtained from the structure of which is 142.7 Hz. In the light of these special spiral-shaped elastic beams, the voltage generated by the piezoelectric effect is up to 29.34 V, which is located in the frequency of 191.7 Hz when vibration energy is transmitted in this structure. Added to this, the quantity of peak output voltage points manufactured is eight. In comparison to the Bragg scatter type defect PnC energy harvesting system proposed by Yang [18], our present system demonstrates a substantial enhancement in performance. Specifically, the peak output voltage has increased by nearly sevenfold, and the peak output electrical energy has surged by approximately ninefold. Furthermore, when contrasted with the locally resonant PnC system featuring spiral beams proposed by Shen [22], our system excels by yielding a significantly higher peak voltage at a comparable operating frequency, showcasing a remarkable improvement of approximately 125.4%. Moreover, the energy recovery system will be prepared for actual experiments in the future.

Through the above study, we have shown that the specific structure has a profound impact on the energy harvesting capabilities of PnCs. Therefore, it provides a novel train of thought regarding the designs of the structures of energy-harvesting PnCs for engineering applications. However, further in-depth works should be carried out to prepare acoustic functional materials, using the 5 × 5 super-cell 3D PnCs presented herein, for possible applications to continuously supply electricity in wireless microsensor systems.

## Figures and Tables

**Table 1:** The mechanical parameters of two materials.

Materials	Density (*ρ*) (kg/m^3^)	Young’s Modulus (*E*) (Pa)	Poisson’s Ratio (*v*)
PA6	1180	2.32 × 10^9^	0.3900
Aluminum metal	11,600	4.08 × 10^10^	0.3691

**Table 2:** Partial parameters of PZT-5H.

Description	Material Parameters		Value
PZT-5H	Elastic compliance	*s*_11_	16.5 pm^2^/N
		*s*_12_	−4.78 pm^2^/N
		*s*_13_	−8.45 pm^2^/N
		*s*_33_	20.7 pm^2^/N
		*s*_44_	43.5 pm^2^/N
		*s*_66_	42.6 pm^2^/N
	Density (*ρ*_P_)		7500 kg/m^3^
	Piezoelectric strain coefficient(*d*_31_)		−274 pm/V
	Piezoelectric constant(*e*_31_)		−6.62 C/m^2^
	Dielectric constant (*ε_T 33_*)		17.29 nF/m^2^

## Data Availability

Data are contained within the article.
